# Remission outcomes in severe eosinophilic asthma with mepolizumab therapy: Analysis of the REDES study

**DOI:** 10.3389/fimmu.2023.1150162

**Published:** 2023-04-12

**Authors:** Ian Pavord, Frances Gardiner, Liam G. Heaney, Christian Domingo, Robert G. Price, Alison Pullan, John Oppenheimer, Guy Brusselle, Hiroyuki Nagase, Geoffrey Chupp, Emilio Pizzichini, David Bañas-Conejero, Peter Howarth

**Affiliations:** ^1^ Respiratory Medicine Unit and Oxford Respiratory National Institute for Health Research Biomedical Research Centre, Nuffield Department of Medicine, University of Oxford, Oxford, United Kingdom; ^2^ Global Medical, Global Specialty and Primary Care Therapy Area, GSK House, Brentford, United Kingdom; ^3^ Wellcome Wolfson Institute for Experimental Medicine, Queen’s University Belfast, Belfast, United Kingdom; ^4^ Servei de Pneumologia, Corporació Sanitària Parc Taulí, Sabadell, Universitat Autònoma de Barcelona (UAB), Barcelona, Spain; ^5^ Biostatistics, GSK, Stevenage, United Kingdom; ^6^ Plus-Project Partnership Ltd, Knutsford, United Kingdom; ^7^ Department of Internal Medicine, Pulmonary and Allergy, UMDNJ-Rutgers New Jersey Medical School, Newark, NJ, United States; ^8^ Department of Respiratory Medicine, Ghent University Hospital, Ghent, Belgium; ^9^ Division of Respiratory Medicine and Allergology, Department of Medicine, Teikyo University School of Medicine, Tokyo, Japan; ^10^ Yale Center for Asthma and Airways Disease (YCAAD), Yale School of Medicine, New Haven, CT, United States; ^11^ Department of Clinical Medicine, Federal University of Santa Catarina, Santa Catarina, Brazil; ^12^ Specialty Care Medical Department, GSK, Madrid, Spain

**Keywords:** severe asthma, remission, clinical outcomes, real-world, eosinophil biology, mepolizumab

## Abstract

**Introduction:**

Clinical remission as a multicomponent treatment goal in severe asthma is being explored in clinical practice. This *post hoc* analysis used data from the REDES study to assess the proportion of patients with severe eosinophilic asthma achieving our multicomponent definitions of clinical remission after 1 year of mepolizumab treatment.

**Methods:**

The real-world, retrospective observational REDES study enrolled patients with severe eosinophilic asthma who were newly prescribed mepolizumab and with ≥12 months of medical records pre-enrolment. Multicomponent clinical remission was defined as: oral corticosteroid (OCS)-free; exacerbation-free; asthma control test (ACT) score ≥20; and with or without post-bronchodilator forced expiratory volume in 1 second ≥80%. Baseline characteristics were also assessed in those who did/did not achieve clinical remission.

**Results:**

37% and 30% of patients with severe eosinophilic asthma met our proposed three- and four-component on-treatment clinical remission definitions; an increase from 2% and 3% at baseline. Most frequently achieved individual components of clinical remission were: OCS-free; ACT score ≥20. For patients fulfilling the multicomponent clinical remission definitions, at baseline we observed higher blood eosinophil counts, better ACT scores and lung function, lower maintenance OCS use, and a slightly lower rate of prior exacerbations versus those who did not.

**Discussion:**

Clinical remission is a realistic target in clinical practice for a subset of patients with severe eosinophilic asthma receiving mepolizumab. Further studies are required to elucidate whether features linked to the underlying endotype can help predict treatment outcomes, increase rates of clinical remission, and potentially modify disease progression.

## 1 Introduction

**Figure 1:**
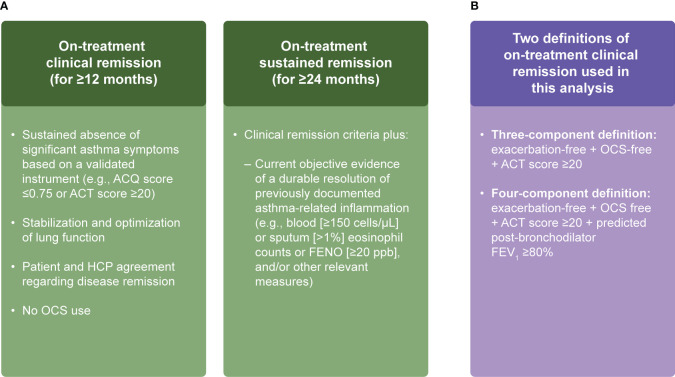
Working definitions of remission in asthma **(A)** and our composite definitions of on-treatment clinical remission for patients with severe asthma **(B)** (3–5). ACQ, Asthma Control Questionnaire; ACT, Asthma Control Test; FENO, fractional exhaled nitric oxide; FEV_1_, forced expiratory volume in 1 second; HCP, healthcare practitioner; OCS, oral corticosteroid; ppb, parts per billion.

In 2018, a *Lancet* Commission paper by Pavord et al. (1) divided asthma management into three eras, the first focusing on bronchodilator use, the second on inhaled corticosteroids use, and the third on precision management (including the use of biologics), and targeted treatment based on a patient’s disease characteristics (**Supplementary Figure 1**). Within the past 2 decades, the advent of biologics has substantially improved the treatment paradigm for diseases including severe asthma (2, 3). The way clinical outcomes are measured in severe asthma is evolving from the assessment of single outcomes to composite outcomes, and for patients with asthma, experts have aimed to reach a consensus on the definition of clinical remission (≥12–<24 months) and sustained remission (≥24 months) for those continuing treatment (**Figure 1A**) (3–5). The definitions include components that represent key clinical criteria and patient-reported outcomes to measure disease activity (3) and allow for the continuation of background therapy as long as patients are not impacted by side effects.

A recent (2022) publication by Menzies-Gow et al. evaluated clinical remission in a subset of patients with severe asthma treated with benralizumab (interleukin-5 [IL-5] receptor α-directed monoclonal antibody) using data from three pivotal Phase III clinical trials (6). This *post hoc* analysis focused only on patients with no baseline oral corticosteroid (OCS) use (selected from SIROCCO/CALIMA) or those receiving <12.5 mg prednisone/prednisolone equivalents/day (ZONDA). The individual components of the composite definition of clinical remission were: no OCS use, no exacerbations, Asthma Control Questionnaire-6 (ACQ-6) score ≤0.75, and pre-bronchodilator forced expiratory volume in 1 second (FEV_1_) increase ≥100 mL. The analysis demonstrated that 14.5% and 22.5% of patients receiving benralizumab achieved remission at 12 (SIROCCO/CALIMA) or 6 months (ZONDA) compared with 7.7% and 7.5% in the respective placebo groups. All trials required patients to remain on background medication with the exception of OCS doses in ZONDA.

Here we sought to further build on the concept of a new era of asthma management, illustrating a potential fourth era, where as a consequence of improved targeted treatment approaches, we envision patients with asthma experiencing better clinical outcomes; namely an era of on-treatment clinical remission (**Supplementary Figure 1**). We performed a *post hoc* analysis using real-world data from the REal worlD Effectiveness and Safety of mepolizumab (REDES) study (7), which included patients with severe eosinophilic asthma who were treated with mepolizumab (anti-IL-5 monoclonal antibody) (8) for 1 year, and applied our two composite clinical remission definitions (OCS-free; exacerbation-free; asthma control test (ACT) score ≥20; and with or without post-bronchodilator FEV_1_ ≥80%). The aim of our analysis was to initiate a dialogue about how biologics might be more imaginatively used, as well as encourage the field to move away from single measures of disease control and to consider using composite measures to assess the goal of clinical remission. This could ultimately lead to the earlier proactive use of biologics to alter disease progression and enable the emergence of an understanding that could herald a fifth era of asthma management, where clinical remission off-treatment would become a possibility allowing patients to lead normal lives with total symptom control.

## 2 Materials and methods

### 2.1 Study design and patients

Details of the REDES study design and patient population have been published previously (7). Briefly, REDES (GSK ID: 213172) was a retrospective, real-world, Phase IV, multicentric, observational cohort study enrolling patients with severe eosinophilic asthma across 24 Spanish hospitals (**Supplementary Figure 2**). The observational period included 12 months pre- and post-mepolizumab treatment. Eligibility criteria for the REDES study included: patients ≥18 years of age with a clinical diagnosis of severe uncontrolled eosinophilic asthma; patients who initiated mepolizumab ≥12 months before the date of inclusion in the study; and had ≥12 months of relevant medical records prior to enrolment. The primary endpoint of the REDES study was the annual rate of clinically significant exacerbations. Secondary endpoints included pre- and post-bronchodilator spirometric tests, and changes in blood eosinophil counts, average OCS daily maintenance dose, and symptom control (ACT score) pre- to post-mepolizumab treatment. These results have been previously reported (7).

### 2.2 Definitions of clinical remission

In this analysis, we used two definitions of on-treatment clinical remission. Firstly, a four-component clinical remission definition that required patients to meet all of the following criteria at Week 52: i) OCS-free; ii) exacerbation-free (for 52 weeks); iii) an ACT score ≥20; and iv) a percent predicted post-bronchodilator FEV_1_ ≥80%. Secondly, a three-component clinical remission definition that included meeting the following criteria at Week 52: i) OCS-free; ii) exacerbation-free (for 52 weeks); and iii) an ACT score ≥20 (**Figure 1B**).

### 2.3 *Post hoc* endpoints included in this analysis

*Post hoc* analyses were performed to determine the proportion of patients meeting the individual components of the clinical remission definitions, to appreciate the individual contribution of each component, and those meeting combinations of these components, including our two definitions of clinical remission at Week 52 (three- and four-component clinical remission). Additionally, a descriptive analysis of differences in the baseline demographics and clinical characteristics of patients according to their remission status at Week 52 (i.e., those who met the clinical remission definitions compared with those who did not) was also performed to gain insight into the responsive population. An exacerbation was defined as the requirement of systemic OCS treatment for ≥3 days, or a doubling of the dose of maintenance OCS, or a visit to an emergency department/hospitalization for treatment.

### 2.4 Statistical analysis

For the description of continuous variables, the mean, median, standard deviation (SD), and interquartile range (IQR) values were used, while categorical variables were described using the number and percentage within categories. All *post hoc* analyses were descriptive in nature and as such no p values were derived.

## 3 Results

### 3.1 Proportion of patients achieving clinical remission

**Figure 2:**
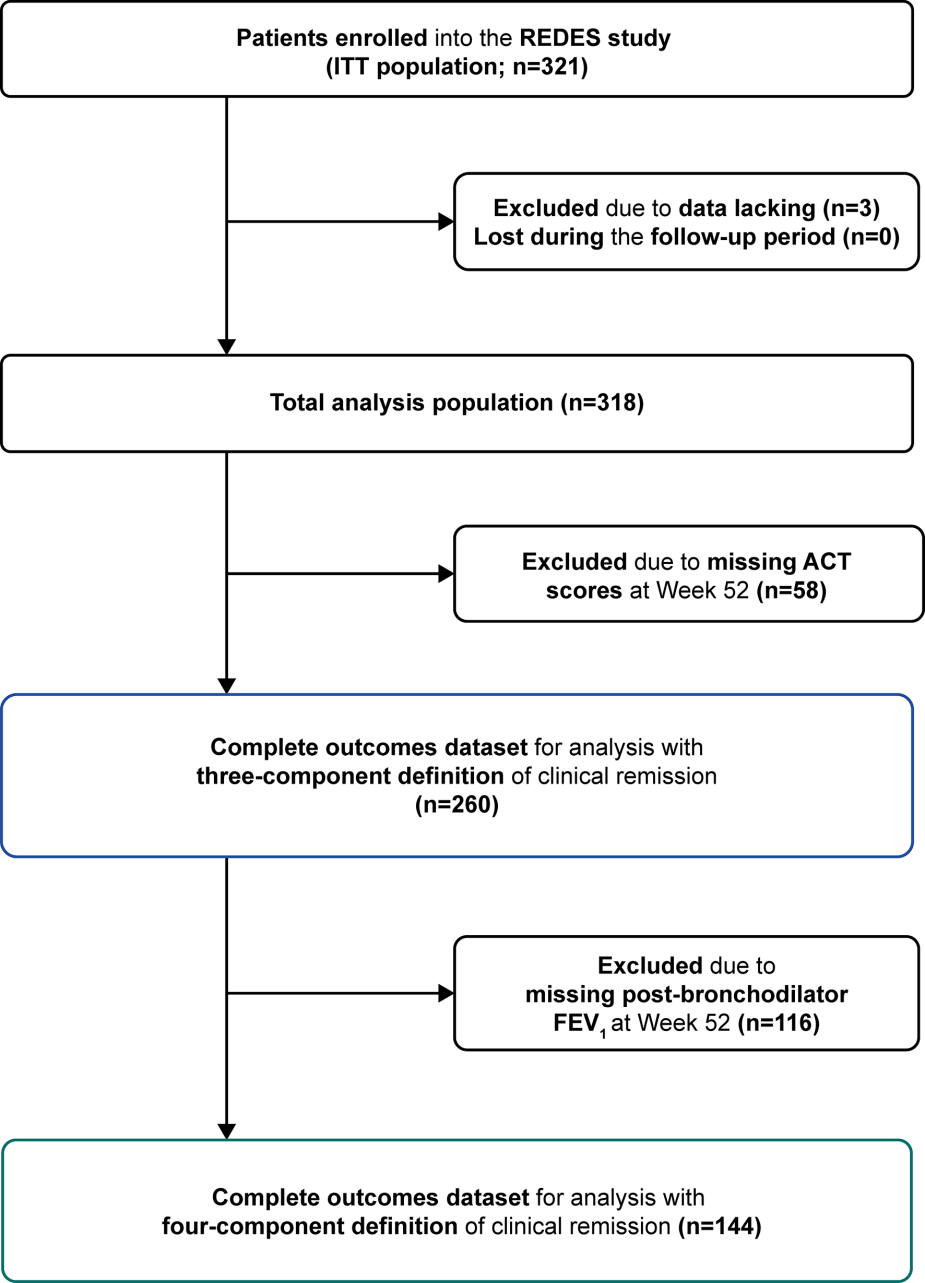
Patient attrition and outcome datasets for analysis. ACT, Asthma Control Test; FEV_1_, forced expiratory volume in 1 second; ITT, intention-to-treat; REDES, REal worlD Effectiveness and Safety of mepolizumab.

Of the 318 patients included in this analysis, data for the three- and four-component clinical remission definitions were available in 260 (82%) and 144 (45%) patients, respectively. Missing post-bronchodilator FEV_1_ or ACT scores at Week 52 accounted for the reduced numbers (**Figure 2**). There were notable differences between the baseline characteristics for the 174 excluded and the 144 included in the four-component definition; for instance, the group with 144 patients had a slightly lower number of exacerbations in the previous year, higher geometric mean blood eosinophil counts, higher ACT scores, and received higher median OCS doses at baseline (**Supplementary Table 1**). Most baseline characteristics for the 58 excluded from the three-component clinical remission definition and the 260 patients included were similar; except that the 260 patients had higher exacerbation rates in the previous year, higher geometric mean blood eosinophil counts, higher ACT scores, and greater prior omalizumab use (**Supplementary Table 1**).

**Figure 3:**
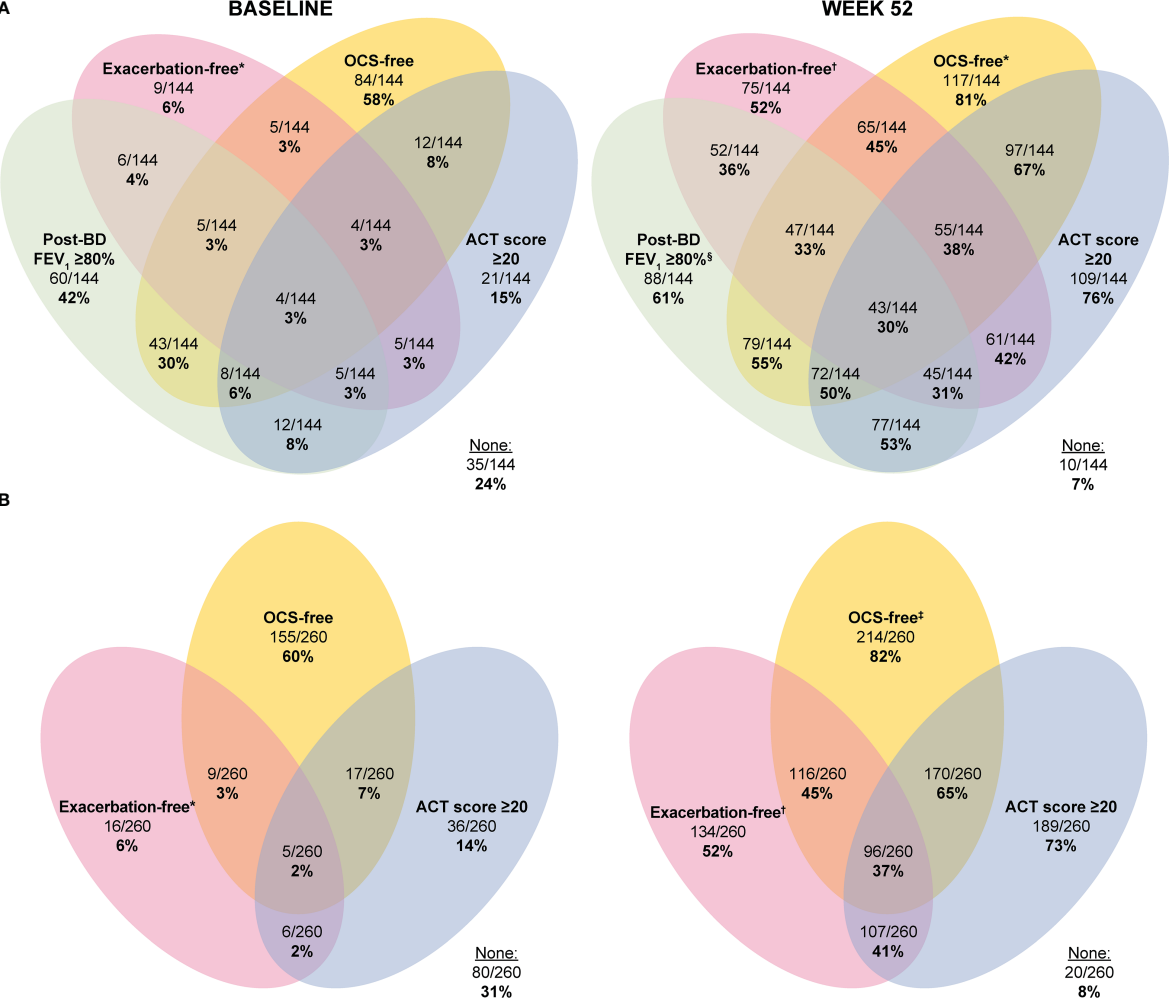
Proportion of patients with severe eosinophilic asthma meeting one or multiple elements of the four- **(A)** and three-component **(B)** clinical remission definitions at baseline and after 1 year of treatment with mepolizumab. In panel A, 174 patients were excluded owing to missing post-bronchodilator FEV_1_ values or ACT scores at Week 52. In Panel B, 58 patients were excluded due to missing ACT scores at Week 52. *No exacerbations experienced in the 12 months prior to baseline (Week 0). ^†^No exacerbations experienced up to study Day 365. ^‡^Patients were not receiving OCS at baseline (Week 0) and remained off OCS at Week 52; ^§^Post-bronchodilator FEV_1_ percent predicted ≥80% at Week 52. ACT, Asthma Control Test; BD, bronchodilator; FEV_1_, forced expiratory volume in 1 second; OCS, oral corticosteroid.

In the 144 patients analyzed, after 1 year of treatment with mepolizumab, 30% fulfilled the four-component clinical remission definition, and 38% fulfilled the three-component definition. This represents an increase (27% and 35%, respectively) from 3% who achieved these criteria at baseline (**Figure 3A**). Moreover, at baseline 3–6% met a combination of three components and 3–30% met a combination of two components of clinical remission. Of those studied 58%, 42%, 15% and 6% of patients met the OCS-free, post-bronchodilator FEV_1_ ≥80%, ACT score ≥20 and exacerbation-free components respectively at baseline. After 1 year of treatment with mepolizumab, 31–50% met the different combinations of three of the components of clinical remission, while 36–67% met two of the components. At least 50% of patients met any individual component of clinical remission; OCS-free (81%) and ACT score ≥20 (76%) were the most frequently achieved components of remission (**Figure 3A**).

In the larger sub-population (n=260), 37% fulfilled the three-component definition of clinical remission after 1 year of mepolizumab treatment, representing an increase (35%) from 2% at baseline (**Figure 3B**). A combination of two of the components of clinical remission was met by 2–7% of patients at baseline, while 60%, 14% and 6% of patients met OCS-free, ACT score ≥20 and exacerbation-free components respectively at baseline. After 1 year of treatment with mepolizumab, 41–65% of patients met a combination of the two different components of clinical remission. At least 50% of patients met any individual component of clinical remission; OCS-free (82%) and ACT score ≥20 (73%) were the most frequently achieved (**Figure 3B**).

### 3.2 Characteristics that provide insight on who may clinically remit

The baseline demographic and clinical characteristics for those who met the three- and four-component clinical remission definitions versus those who did not are presented in **Tables 1**, **2**.

**Table 1:** Patient baseline demographics and clinical characteristics in those who did/did not meet the four-component definition of clinical remission following 1 year of mepolizumab treatment.

	Four-component clinical remission definition*	
	Patients who achieved clinical remission (n=43)	Patients who did not achieve clinical remission (n=101)
**Age, mean (SD), years**	58.1 (9.06)	58.8 (14.17)
**Age at asthma diagnosis, mean (SD), years**	n=43; 32.2 (16.62)	n=99; 34.3 (18.99)
**Female, n (%)**	35 (81)	73 (72)
**Ethnicity, n (%)**		
Caucasian	39 (91)	93 (92)
Hispanic	4 (9)	6 (6)
African	0	2 (2)
Other	0	0
**BMI, mean (SD), kg/m^2^**	n=42; 29.0 (5.15)	n=101; 27.8 (5.19)
**BMI category, n (%), kg/m^2^**	n=42	n=101
<18.5	0	1 (<1)
18.5–<25.0	11 (26)	28 (28)
25.0–<30.0	14 (33)	46 (46)
30.0–<35.0	13 (31)	17 (17)
≥35.0	4 (10)	9 (9)
**Smoking status, n (%)**		
Never	26 (60)	60 (59)
Ex-smoker (>6 months)	16 (37)	39 (39)
Current smoker	0	0
Passive smoker	1 (2)	1 (<1)
Not available	0	1 (<1)
**Exacerbations in the 12 months pre-mepolizumab treatment, mean (SD)**	4.1 (3.25)	4.2 (3.10)
**Baseline blood eosinophil count,** ^†^ **geometric mean (SD log), cells/µL**	653.85 (0.546)	504.22 (0.779)
**Baseline blood eosinophil count category,^†^ n (%), cells/µL**		
<150	1 (2)	7 (7)
150–<300	3 (7)	9 (9)
300–<500	5 (12)	25 (25)
500–<700	10 (23)	23 (23)
≥700	24 (56)	37 (37)
**Allergic asthma, n (%)**	25 (58)	51 (50)
**Atopic sensitization, n (%)**	n=43; 14 (33)	n=101; 43 (43)
**Baseline ACT score, mean (SD)**	n=41; 15.9 (4.88)	n=95; 13.7 (4.99)
**Baseline ACT score category, n (%)**	n=41	n=95
<20 (uncontrolled)	31 (76)	84 (88)
≥20 (controlled)	10 (24)	11 (12)
**Baseline post-bronchodilator FEV_1_%pred, mean (SD)**	n=41 86.9 (21.52)	n=88 71.2 (23.39)
**OCS dependent in the 12 months pre-mepolizumab treatment, n (%)**	7 (16)	53 (52)
**Baseline OCS dose, median (IQR), mg/day**	n=3; 6.3 (5.0, 15.0)	n=42; 10.0 (5.0, 15.0)
**Previous omalizumab treatment, n (%)**	n=43; 11 (26)	n=101; 41 (41)
**Comorbidities, n (%)**		
Anxiety	12 (28)	21 (21)
Atopic dermatitis	0	2 (2)
Bronchiectasis	10 (23)	26 (26)
Depression	5 (12)	24 (24)
EGPA	1 (2)	5 (5)
GERD	9 (21)	26 (26)
Hypersensitivity to NSAIDs	8 (19)	11 (11)
CRSwNP	20 (47)	44 (44)

*Definition of clinical remission: exacerbation-free for 52 weeks, OCS-free at Week 52, ACT score ≥20 at Week 52, and predicted post-bronchodilator FEV_1_ ≥80% at Week 52. 174 patients were excluded owing to missing post-bronchodilator FEV_1_ values or ACT scores at Week 52. ^†^Where a blood eosinophil count of zero was recorded, a small value (i.e., minimum all non-missing results/2) was added prior to log transformation. ACT, asthma control test; BMI, body mass index; CRSwNP, chronic rhinosinusitis with nasal polyps; EGPA, eosinophilic granulomatosis with polyangiitis; FEV_1_, forced expiratory volume in 1 second; GERD, gastroesophageal reflux disease; IQR, interquartile range; NSAID, non-steroidal anti-inflammatory drug; OCS, oral corticosteroid; pred, predicted; SD, standard deviation.

**Table 2:** Patient baseline demographics and clinical characteristics in those who did/did not meet the three-component definition of clinical remission following 1 year of mepolizumab treatment.

	Three-component clinical remission definition^†^	
	Patients who achieved clinical remission (n=96)	Patients who did not achieve clinical remission (n=164)
**Age, mean (SD), years**	56.9 (10.30)	57.0 (14.07)
**Age at asthma diagnosis, mean (SD), years**	n=96; 32.2 (16.52)	n=155; 35.5 (18.85)
**Female, n (%)**	70 (73)	120 (73)
**Ethnicity, n (%)**		
Caucasian	88 (92)	147 (90)
Hispanic	8 (8)	13 (8)
African	0	3 (2)
Other	0	1 (<1)
**BMI, mean (SD), kg/m^2^**	n=95; 28.0 (5.03)	n=164; 28.6 (5.69)
**BMI category, n (%), kg/m^2^**	n=95	n=164
<18.5	0	1 (<1)
18.5–<25.0	31 (33)	41 (25)
25.0–<30.0	33 (35)	69 (42)
30.0–<35.0	23 (24)	35 (21)
≥35.0	8 (8)	18 (11)
**Smoking status, n (%)**		
Never	63 (66)	100 (61)
Ex-smoker (>6 months)	31 (32)	54 (33)
Current smoker	0	2
Passive smoker	1 (1)	5 (3)
Not available	1 (1)	3 (2)
**Exacerbations in the 12 months pre-mepolizumab treatment, mean (SD)**	4.1 (3.34)	4.9 (3.61)
**Baseline blood eosinophil count,** ^†^ **geometric mean (SD log), cells/µL**	675.94 (0.693)	433.24 (1.521)
**Baseline blood eosinophil count category,^†^ n (%), cells/µL**		
<150	3 (3)	13 (8)
150–<300	7 (7)	13 (8)
300–<500	15 (16)	48 (29)
500–<700	21 (22)	35 (21)
≥700	50 (52)	55 (34)
**Allergic asthma, n (%)**	55 (57)	103 (63)
**Atopic sensitization, n (%)**	n=96; 33 (34)	n=163; 75 (46)
**Baseline ACT score, mean (SD)**	n=90; 15.2 (5.04)	n=154; 13.6 (4.93)
**Baseline ACT score category, n (%)**	n=90	n=154
<20 (uncontrolled)	72 (80)	136 (88)
≥20 (controlled)	18 (20)	18 (12)
**Baseline post-bronchodilator FEV_1_%pred, mean (SD)**	n=70 82.2 (23.29)	n=102 73.6 (22.66)
**OCS dependent in the 12 months pre-mepolizumab treatment, n (%)**	20 (21)	85 (52)
**Baseline OCS dose, median (IQR), mg/day**	n=13; 5.0 (4.0, 13.3)	n=67; 10.0 (5.0, 20.0)
**Previous omalizumab treatment, n (%)**	n=96; 34 (35)	n=163; 71 (44)
**Comorbidities, n (%)**		
Anxiety	17 (18)	34 (21)
Atopic dermatitis	1 (1)	1 (<1)
BronchiectasisP	19 (20)	33 (20)
Depression	10 (10)	37 (23)
EGPA	2 (2)	7 (4)
GERD	15 (16)	43 (26)
Hypersensitivity to NSAIDs	18 (19)	19 (12)
CRSwNP	54 (56)	67 (41)

*Definition of clinical remission: exacerbation-free for 52 weeks, OCS-free at Week 52, and ACT score ≥20 at Week 52. 58 patients were excluded with missing ACT score at Week 52. ^†^Where a blood eosinophil count of zero was recorded, a small value (i.e., minimum all non-missing results/2) was added prior to log transformation. ACT, asthma control test; BMI, body mass index; CRSwNP, chronic rhinosinusitis with nasal polyps; EGPA, eosinophilic granulomatosis with polyangiitis; FEV_1_, forced expiratory volume in 1 second; GERD, gastroesophageal reflux disease; IQR, interquartile range; NSAID, non-steroidal anti-inflammatory drug; OCS, oral corticosteroid; pred, predicted; SD, standard deviation.

Results demonstrated that patients who fulfilled both the three- and four-component definitions had higher geometric mean blood eosinophil counts, better ACT scores, and lower maintenance OCS use compared with those who did not meet each of these definitions. For the patients who did not meet the three- or four-component clinical remission definitions, a slightly higher exacerbation rate in the prior year and worse baseline lung function was seen compared with patients who met either definition. In those fulfilling both clinical remission definitions, patients were less likely to have depression, atopic sensitization, or gastroesophageal reflux disease at baseline, and more likely to have hypersensitivity to non-steroidal anti-inflammatory drugs and chronic rhinosinusitis with nasal polyps (CRSwNP) than those not achieving each composite definition. Patients who met the four-component definition were more likely to have allergic asthma compared with those who did not meet this definition; this was in contrast to those meeting the three-component definition, where allergic asthma was less frequently observed compared with those not meeting the definition.

## 4 Discussion

Using our proposed multicomponent definitions of clinical remission, which were aligned with the prior definitions put forward based on an expert consensus collected by a Delphi survey (3, 4), we identified that a subset of patients (37% and 30%) with severe eosinophilic asthma met our proposed three- and four-component definitions of clinical remission following 1 year of mepolizumab treatment. These results demonstrate that on-treatment clinical remission is a realistic target in severe asthma for those treated with mepolizumab. To our knowledge, this is the largest study reporting real-world data on remission outcomes for patients with severe asthma using multicomponent definitions of clinical remission. This is important as it is recognized that approximately 80% of patients with severe eosinophilic asthma would be excluded from enrolling in randomized controlled trials, owing to the rigorous eligibility criteria, despite being sufficiently severe and biologically suitable for anti-eosinophilic biologics (9). As such, remission outcomes generated from registration trial data have limited applicability to outcomes in standard clinical care. Other studies have focused on assessing a patient’s level of treatment response to identify responders and super-responders, such as the RELIght study, a real-world study in which patients with severe eosinophilic asthma were treated with mepolizumab (10). A recent Delphi consensus defined the criteria for identifying super-responders as patients who had achieved dramatic improvements in at least three or more domains after 1 year of treatment (11). Our analysis of real-world outcomes in a biologically relevant, but otherwise unrestricted, severe asthma population not only identifies the proportion of patients achieving clinical remission based on our composite three- and four-component definitions, but also helps to understand the responsiveness of the individual components at this stage of disease severity in patients receiving mepolizumab treatment. Improvements in asthma control, as reflected by the ACT score, and OCS-freedom (no exacerbation bursts and no maintenance therapy) were the most commonly achieved individual components of clinical remission.

The real-world REDES study (7) was selected to assess clinical remission in a real-world severe asthma population, as it had the most complete dataset available to us and represented a severe asthma population that was clinically relevant, with a broad spectrum of comorbidities and a significant unmet need as far as asthma control was concerned. In REDES, for patients who had data available on ACT score (n=279), most patients (85%; n=237) had poor asthma control (ACT score <20) at baseline; and of the 318 included in the study, the majority of patients (92%; n=292) had experienced a severe OCS-requiring exacerbation in the 12 months prior to treatment and 92% had ≥1 comorbidity (n=292). Moreover, patients had a geometric mean blood eosinophil count of 470 cells/µL at baseline. These results demonstrate the severity of disease in this severe eosinophilic asthma population despite receiving standard-of-care treatment in line with the Global Initiative for Asthma (GINA) recommendations up to the initiation of biologic therapy (12) and also reflect the nature of severe eosinophilic asthma managed in routine clinical care.

In the sentinel publication by Menzies-Gow et al (4) the authors initiated a conversation about remission outcomes in severe asthma populations and proposed a definition of remission, based on a Delphi consensus and by using learnings from other chronic inflammatory diseases treated with biologics. Their definition of remission used optimization and stabilization of lung function as one of the criteria (4). To date, no consensus definition for improvements in FEV_1_ have been established. In our analysis, we have taken the ambitious definition of achieving ≥80% predicted post-bronchodilator FEV_1_ as our reflection of optimization of lung function, as this is what could be classified as within the normal range. A recently published (2023) observational multicenter retrospective study by Maglio et al (13), also used ≥80% predicted post-bronchodilator FEV_1,_ alongside OCS-free, exacerbation-free and ACT score ≥20, aligning with our four-component definition of remission. In their study, in 83 patients with severe asthma treated with mepolizumab for 12 months, they reported the same proportion of patients (30%) achieving remission as reported here using our four-way definition. When comparing the proportion of patients achieving the individual components of remission, differences were seen between the two studies. In another *post hoc* analysis, data from the LIBERTY ASTHMA QUEST study in patients with uncontrolled, moderate-to-severe asthma demonstrated that 20.1% (n=70) of patients who received dupilumab (anti-IL-4 receptor α-directed biologic) achieved a three-component definition of asthma remission following 1 year of treatment (no exacerbations, ACQ-5 total score <1.5 and post-bronchodilator FEV_1_ >80%) versus 4.6% (n=9) in the placebo group (14). However, it is well recognized that patients with chronic asthma, particularly severe asthma, have sub-optimal lung function with some showing limited reversibility (9, 15), potentially attributable to structural airway remodeling (16, 17) or impaired lung growth during infancy and adolescence (18, 19). As such, in many patients with severe asthma it would be unrealistic to achieve normal lung function, and it may be more appropriate to evaluate lung function on a case-by-case basis depending on the disease severity, and aim for stabilization with no further decline. Indeed, by analogy, a pragmatic approach was taken with rheumatoid arthritis in which, rather than the structural deformity being expected to resolve, the prevention of disease progression and improvement in functionality were the initial targets when assessing the impact of biologic therapy (20). This has further developed, following evidence of disease modification, to biologics being used early in the course of the disease to prevent the development of structural damage (21, 22). If prevention of disease progression was applied to the lung function outcome measures in REDES, after 1 year of mepolizumab treatment, 63% (n=79/125) of patients would qualify as having no worsening from baseline in post-bronchodilator FEV_1_. Several factors have been linked to an accelerated lung function decline, such as severe exacerbations, increased blood eosinophil counts, elevated fractional exhaled nitric oxide (FeNO) levels, and exposure to environmental factors (e.g., tobacco smoking) (23–25). In the REDES study, mepolizumab reduced exacerbations and eosinophilic inflammation, two of the recognized adverse factors for poorer lung function outcomes (7). Four (1%) patients were current smokers with a mean (SD) pack year history of 20 (10.2), while 33% (n=106) were ex-smokers, and it has been shown that decline in lung function can still progress in smokers despite smoking cessation (26). Further support for the use of mepolizumab to prevent lung function decline has been demonstrated by a Belgian severe asthma registry publication, which reported that treatment with anti-IL-5 biologic therapy as part of standard clinical care attenuated lung function decline in patients with severe asthma (27).

For this clinical remission analysis, we did not select randomized controlled trials to assess clinical remission for several reasons. Although well-structured for gathering data, randomized controlled trials typically reflect a narrow patient population compared with the real world owing to the stringent eligibility criteria. In addition, treatment is also often fixed meaning it is not possible to assess OCS reduction, unless it is a study focusing on OCS tapering. As such, in a recent analysis of remission outcomes from the benralizumab SIROCCO and CALIMA registration studies in severe eosinophilic asthma, patients entering on maintenance OCS were excluded from the analysis. Despite this strongly favoring a positive 1-year outcome for the remission criterium of no maintenance OCS, using a four-component analysis (no OCS, no exacerbations, ACQ-6 score ≤0.75, and pre-bronchodilator FEV_1_ increase ≥100 mL) they reported that 14.5% (n=85/586) of patients treated with benralizumab fulfilled the remission definition at 12 months, compared with 7.7% (n=48/620) of patients treated with placebo (6). No such restriction was applied to the remission analysis within the REDES dataset, in which 31% (n=98/318) were on maintenance OCS therapy at mepolizumab initiation. In relation to the lung function criteria, a 100 mL improvement in FEV_1_ at either 6 or 12 months has since been criticized as a criterium for the assessment of remission, as the definition originally proposed by the same leading author was only to be undertaken at 12 months and did not define a 100 mL improvement as a valid outcome (28). These data underscore the need to better understand whether lung function fits within a definition of remission in severe asthma or whether it is a disease modification validation outcome measure, used to assess the value of remission achieving therapy. We would therefore advocate for the exclusion of lung function parameters from a clinical remission definition until further data are generated that inform the position, and instead suggest utilization of the more accepted three-component definition discussed here as a starting point. Alternatively, we would support that prevention of decline in lung function, encompassing stabilization and improvement, is used in lieu of focusing solely on improvements in lung function.

The determination of demographic or clinical markers that can predict whether a patient may or may not achieve clinical remission would be of great benefit. Our analyses suggest that at baseline those patients who achieved our measures of clinical remission (three- and four-component definitions) had higher blood eosinophil counts, better ACT scores and lung function, lower maintenance OCS use, and a slightly lower rate of prior exacerbations compared with those who did not. Patients meeting the three- and four-component definitions were more likely to be non-atopic and have CRSwNP versus those who did not achieve remission (although for the latter the differences were small in the four-component remission dataset); the higher likelihood of the presence of CRSwNP may suggest that these subgroups were more likely to have type-2-driven disease, potentially explaining the better chance that they would reach clinical remission on therapy with mepolizumab (29). An alternative explanation would be that a patient’s chance of achieving remission depends on their baseline disease severity and those not achieving clinical remission relied on higher maintenance OCS doses, which may have led to the lower blood eosinophil counts seen in those not achieving clinical remission. Patients who achieved clinical remission were also less likely to suffer from depression or gastroesophageal reflux. It has been reported that these comorbidities make it less likely for patients to achieve substantial improvements in asthma control as they may, in addition to the underlying asthma, contribute to the level of symptom reporting (30). It is therefore understandable that as symptoms, such as breathlessness, are not specific to asthma, they may limit the likelihood of achieving a remission-defined level of control. Overall, these data support that initiating treatment at an earlier stage in disease development, in order to prevent the development of severe disease (and associated conditions) or disease worsening, may lead to better long-term outcomes for patients; and this will require further studies.

There were several limitations of this study. First, as to be expected for a real-world study, data gathering was retrospective and opportunistic, and as such complete datasets were not available on all participants. The most limited outcome data in this study population was the post-bronchodilator FEV_1_ and this may be in part owing to the 2019 coronavirus disease pandemic disrupting clinical appointments. As a result, assessment of the different definitions of clinical remission had to be analyzed within reduced outcome datasets (as per **Figure 2**), where the applicable component data were available and collected at Week 52. There were some notable differences between the baseline characteristics for the patients excluded versus included for analysis with the three- and four-component definitions. While this may infer a selection bias, the potential link with asthma severity was not consistent, and therefore we believe our results should be generalizable to the wider severe asthma population. This analysis was based on an open-label study without a placebo arm and used pre-treatment (baseline) as a comparison. Moreover, the *post hoc* nature of the analysis means care must be taken in data interpretation as the analysis may not be powered to optimally answer the questions being asked, with a risk of type I error and overinterpretation. As mentioned previously, this is an exploratory application of a definition for clinical remission, which will require revision over time as further data become available. Nonetheless, it does provide valuable insight into the performance of different components of remission within a real-world severe asthma population. There is a clear need for a more unified approach to the utilization of lung function outcomes and recognition that while patient-reported outcome measures, such as ACT score, are essential components, they also have limitations. For example, some of the scoring components are not specific for asthma and those related to comorbidities not targeted by specific asthma interventions will remain. This implies that there may be a need for a multi-targeted approach addressing a range of treatable traits to gain the optimum outcome for each patient. In addition, consideration should be given to the adoption of newer measures that include additional parameters such as future risk of exacerbations (e.g., Asthma Impairment and Risk Questionnaire, which is currently being validated) (31). Furthermore, as this study was based on data from patients with asthma from a single country, there may be variations compared with other patient populations that are not reflected within this analysis. Finally, longer-term studies (≥2 years) are required to assess whether sustained remission while on treatment with mepolizumab is achievable.

## 5 Conclusions

The results of this *post hoc* analysis provide evidence that, in daily clinical practice, mepolizumab treatment enables a subset of patients with severe eosinophilic asthma to achieve the proposed composite definitions of clinical remission, with more than one-third meeting the three-component definition. Baseline clinical characteristics including specific comorbidities that are linked to the underlying endotype and more commonly associated with clinical remission achievement may help to predict patients who are more likely to respond to mepolizumab treatment and reach greater degrees of long-term disease control. The inclusion of clinical remission criteria within future treatment plans, a focus on intervening earlier, and being able to predict the patients who are most likely to achieve remission will help optimize patient care. These changes will also allow personalized approaches to treatment and ultimately, help us progress into the fourth era of asthma management and beyond.

## Data availability statement

The original contributions presented in the study are included in the article/**Supplementary Material**. Further inquiries can be directed to the corresponding author.

## Ethics statement

The REDES study (GSK ID: 213172), which involved human participants was reviewed and approved by Hospital La Princesa, Madrid.

## Author contributions

PH and DB-C were involved in the conception and design of the work**;** CD was involved with the acquisition of data; all authors were involved in drafting the work or revising it critically for important intellectual content (i.e., data analysis and interpretation) and all authors agreed to the submission and to be accountable for all aspects of the work. All authors contributed to the article and approved the submitted version.

## References

[1] Pavord ID Beasley R Agusti A Anderson GP Bel E Brusselle G. After asthma: redefining airways diseases. Lancet (2018) 391:350–400. doi: 10.1016/S0140-6736(17)30879-6 28911920

[2] Kardas G Kuna P Panek M. Biological therapies of severe asthma and their possible effects on airway remodeling. Front Immunol (2020) 11:1134. doi: 10.3389/fimmu.2020.01134 32625205 PMC7314989

[3] Menzies-Gow A Szefler SJ Busse WW. The relationship of asthma biologics to remission for asthma. J Allergy Clin Immunol Pract (2021) 9:1090–8. doi: 10.1016/j.jaip.2020.10.035 33130146

[4] Menzies-Gow A Bafadhel M Busse WW Casale TB Kocks JWH Pavord ID. An expert consensus framework for asthma remission as a treatment goal. J Allergy Clin Immunol (2020) 145:757–65. doi: 10.1016/j.jaci.2019.12.006 31866436

[5] Agache I Akdis CA Akdis M Canonica GW Casale T Chivato T. EAACI biologicals guidelines-recommendations for severe asthma. Allergy (2021) 76:14–44. doi: 10.1111/all.14425 32484954

[6] Menzies-Gow A Hoyte FL Price DB Cohen D Barker P Kreindler J. Clinical remission in severe asthma: a pooled *post hoc* analysis of the patient journey with benralizumab. Adv Ther (2022) 39:2065–84. doi: 10.1007/s12325-022-02098-1 PMC9056458 35287231

[7] Domingo Ribas C Carrillo Diaz T Blanco Aparicio M Martinez Moragon E Banas Conejero D Sanchez Herrero MG. REal worlD effectiveness and safety of mepolizumab in a multicentric Spanish cohort of asthma patients stratified by eosinophils: the REDES study. Drugs (2021) 81:1763–74. doi: 10.1007/s40265-021-01597-9 PMC8550660 34586602

[8] Mepolizumab (Nucala) prescribing information (2022). Available at: https://gskpro.com/content/dam/global/hcpportal/en_US/Prescribing_Information/Nucala/pdf/NUCALA-PI-PIL-IFU-COMBINED.PDF.

[9] Brown T Jones T Gove K Barber C Elliott S Chauhan A. Randomised controlled trials in severe asthma: selection by phenotype or stereotype. Eur Respir J (2018) 52. doi: 10.1183/13993003.01444-2018 30361247

[10] Kallieri M Zervas E Fouka E Porpodis K Mitrova MH Tzortzaki E. RELIght: A two-year REal-LIfe study of mepolizumab in patients with severe eosinophilic asTHma in Greece: Evaluating the multiple components of response. Allergy (2022) 77:2848–52. doi: 10.1111/all.15382 35595723

[11] Upham JW Le Lievre C Jackson DJ Masoli M Wechsler ME Price DB. Defining a severe asthma super-responder: Findings from a Delphi process. J Allergy Clin Immunol Pract (2021) 9:3997–4004. doi: 10.1016/j.jaip.2021.06.041 34271216

[12] Global strategy for asthma management and prevention (2022). Available at: https://ginasthma.org/wp-content/uploads/2022/05/GINA-Main-Report-2022-FINAL-22-05-03-WMS.pdf.

[13] Maglio A Vitale C Pelaia C D'Amato M Ciampo L Sferra E. Severe asthma remissions induced by biologics targeting IL5/IL5r: Results from a multicenter real-life study. Int J Mol Sci (2023) 24. doi: 10.3390/ijms24032455 PMC9916787 36768778

[14] Pavord I Busse W Israel E Szefler S Chen Z. Dupilumab treatment leads to clinical asthma remission in patients with uncontrolled moderate-to-severe asthma with type 2 inflammation. J Allergy Clin Immunol (2021) 147, Supplement AB4. doi: 10.1016/j.jaci.2020.12.061

[15] Moore WC Bleecker ER Curran-Everett D Erzurum SC Ameredes BT Bacharier L. Characterization of the severe asthma phenotype by the national heart, lung, and blood institute's severe asthma research program. J Allergy Clin Immunol (2007) 119:405–13. doi: 10.1016/j.jaci.2006.11.639 PMC2837934 17291857

[16] Niimi A Matsumoto H Amitani R Nakano Y Mishima M Minakuchi M. Airway wall thickness in asthma assessed by computed tomography. relation to clinical indices. Am J Respir Crit Care Med (2000) 162:1518–23. doi: 10.1164/ajrccm.162.4.9909044 11029371

[17] Kasahara K Shiba K Ozawa T Okuda K Adachi M. Correlation between the bronchial subepithelial layer and whole airway wall thickness in patients with asthma. Thorax (2002) 57:242–6. doi: 10.1136/thorax.57.3.242 PMC1746264 11867829

[18] McGeachie MJ Yates KP Zhou X Guo F Sternberg AL Van Natta ML. Patterns of growth and decline in lung function in persistent childhood asthma. N Engl J Med (2016) 374:1842–52. doi: 10.1056/NEJMoa1513737 PMC5032024 27168434

[19] Hough KP Curtiss ML Blain TJ Liu RM Trevor J Deshane JS. Airway remodeling in asthma. Front Med (Lausanne) (2020) 7:191. doi: 10.3389/fmed.2020.00191 32509793 PMC7253669

[20] Curtis JR Singh JA. Use of biologics in rheumatoid arthritis: current and emerging paradigms of care. Clin Ther (2011) 33:679–707. doi: 10.1016/j.clinthera.2011.05.044 21704234 PMC3707489

[21] Monti S Montecucco C Bugatti S Caporali R. Rheumatoid arthritis treatment: the earlier the better to prevent joint damage. RMD Open (2015) 1:e000057. doi: 10.1136/rmdopen-2015-000057 26557378 PMC4632152

[22] Smolen JS Landewé RBM Bijlsma JWJ Burmester GR Dougados M Kerschbaumer A. EULAR recommendations for the management of rheumatoid arthritis with synthetic and biological disease-modifying antirheumatic drugs: 2019 update. Ann Rheum Dis (2020) 79:685–99. doi: 10.1136/annrheumdis-2019-216655 31969328

[23] Ortega H Yancey SW Keene ON Gunsoy NB Albers FC Howarth PH. Asthma exacerbations associated with lung function decline in patients with severe eosinophilic asthma. J Allergy Clin Immunol Pract (2018) 6:980–6.e1. doi: 10.1016/j.jaip.2017.12.019 29398640

[24] Graff S Demarche S Henket M Paulus V Louis R Schleich F. Increase in blood eosinophils during follow-up is associated with lung function decline in adult asthma. Respir Med (2019) 152:60–6. doi: 10.1016/j.rmed.2019.04.020 31128611

[25] Coumou H Westerhof GA de Nijs SB Zwinderman AH Bel EH. Predictors of accelerated decline in lung function in adult-onset asthma. Eur Respir J (2018) 51:1701785. doi: 10.1183/13993003.01785-2017 29444915

[26] Oelsner EC Balte PP Bhatt SP Cassano PA Couper D Folsom AR. Lung function decline in former smokers and low-intensity current smokers: a secondary data analysis of the NHLBI pooled cohorts study. Lancet Respir Med (2020) 8:34–44. doi: 10.1016/S2213-2600(19)30276-0 31606435 PMC7261004

[27] Graff S Brusselle G Hanon S Sohy C Dupont L Peche R. Anti-interleukin-5 therapy is associated with attenuated lung function decline in severe eosinophilic asthma patients from the Belgian severe asthma registry. J Allergy Clin Immunol Pract (2022) 10:467–77. doi: 10.1016/j.jaip.2021.09.023 34563736

[28] Calzetta L Rogliani P. Letter to the Editor regarding "Clinical remission in severe asthma: A pooled *Post hoc* analysis of the patient journey with benralizumab". Adv Ther (2022) 39:3857–61. doi: 10.1007/s12325-022-02213-2 PMC9309134 35731338

[29] Price D Menzies-Gow A Bachert C Canonica GW Kocks J Khan AH. Association between a type 2 inflammatory disease burden score and outcomes among patients with asthma. J Asthma Allergy (2021) 14:1173–83. doi: 10.2147/JAA.S321212 PMC8488033 34616157

[30] Harvey ES Langton D Katelaris C Stevens S Farah CS Gillman A. Mepolizumab effectiveness and identification of super-responders in severe asthma. Eur Respir J (2020) 55:1902420. doi: 10.1183/13993003.02420-2019 32139455

[31] Murphy KR Chipps B Beuther DA Wise RA McCann W Gilbert I. Development of the asthma impairment and risk questionnaire (AIRQ): a composite control measure. J Allergy Clin Immunol Pract (2020) 8:2263–74.e5. doi: 10.1016/j.jaip.2020.02.042 32387166

