# Supplementary material for: Remission outcomes in severe eosinophilic asthma with mepolizumab therapy: Analysis of the REDES study
Source: Front Immunol. 2023 Apr 12;14:1150162. doi: 10.3389/fimmu.2023.1150162 (PMC10131245; doi:10.3389/fimmu.2023.1150162)
Supplement: Supplementary file 1 [file DataSheet_1.docx]

## Supplementary Materials

**Contents**

**Methods.** Additional information on the post hoc endpoints.

**Supplementary Figure 1.** Eras of asthma management and goals.^1,3,12^

**Supplementary Figure 2.** Study design.

**Supplementary Table 1.** Patient baseline demographics and clinical characteristics in the complete outcomes datasets and in the datasets with missing predicted post-bronchodilator FEV_1_ ≥80% and/or missing ACT score.

**Methods.** Additional information on the post hoc endpoints.

An on-treatment strategy was applied to the intercurrent event of the early discontinuation of the investigational drug product. Patients who discontinued mepolizumab treatment prior to Week 52, who had concurrent treatment with another biologic (other than mepolizumab) at Week 52, or had missing responses in any individual criteria were considered as failing to meet the criteria in question. The collection of exacerbations and concomitant OCS use were available across all participants; however, all other assessments were only collected when performed within local clinical practice. As a result, separate complete outcomes datasets were defined for the assessment of the three- and four-component clinical remission definitions.

**Supplementary Table 1.** Patient baseline demographics and clinical characteristics in the complete outcomes datasets and in the datasets with missing predicted post-bronchodilator FEV_1_ ≥80% and/or missing ACT score.

|  | **Complete outcomes dataset (for analysis using the four-component clinical remission definition)**  **n=144** | **Dataset with missing ACT score and predicted post-bronchodilator FEV_1_ ≥80% at Week 52**  **n=174** | **Complete outcomes dataset (for analysis using the three-component clinical remission definition)**  **n=260** | **Dataset with missing ACT score at Week 52**  **n=58** |
| --- | --- | --- | --- | --- |
| **Age, mean (SD), years** | 58.6 (12.83) | 54.9 (11.98) | 57.0 (12.79) | 54.6 (10.97) |
| **Age at asthma diagnosis, mean (SD), years** | n=142  33.7 (18.27) | n=159  34.4 (17.61) | n=251  34.3 (18.03) | n=50  33.1 (17.36) |
| **Female, n (%)** | 108 (75) | 112 (64) | 190 (73) | 30 (52) |
| **Ethnicity, n (%)**  Caucasian  Hispanic  African  Other | 132 (92)  10 (7)  2 (1)  0 | 151 (87)  21 (12)  1 (<1)  1 (<1) | 235 (90)  21 (8)  3 (1)  1 (<1) | 48 (83)  10 (17)  0  0 |
| **BMI, mean (SD), kg/m^2^** | n=143 28.2 (5.18) | n=173  28.9 (5.73) | n=259  28.3 (5.45) | n=57  29.7 (5.60) |
| **BMI category, n (%), kg/m^2^**  <18.5  18.5–<25.0  25.0–<30.0  30.0–<35.0  ≥35.0 | n=143  1 (<1)  39 (27)  60 (42)  30 (21)  13 (9) | n=173  1 (<1)  45 (26)  62 (36)  42 (24)  23 (13) | n=259  1 (<1)  72 (28)  102 (39)  58 (22)  26 (10) | n=57  1 (2)  12 (21)  20 (35)  14 (25)  10 (18) |
| **Smoking status, n (%)**  Never  Ex-smoker (>6 months)  Current smoker  Passive smoker  Not available | 86 (60)  55 (38)  0  2 (1)  1 (<1) | 112 (64)  51 (29)  4 (2)  4 (2)  3 (2) | 163 (63)  85 (33)  2 (<1)  6 (2)  4 (2) | 35 (60)  21 (36)  2 (3)  0  0 |
| **Exacerbations in the 12 months pre-mepolizumab treatment, mean (SD)** | 4.2 (3.13) | 4.7 (3.79) | 4.6 (3.52) | 3.9 (3.44) |
| **Baseline blood eosinophil count,*** **geometric mean (SD log), cells/µL** | n=144  544.91 (0.725) | n=173  415.07 (1.751) | n=260  510.57 (1.296) | n=57  320.99 (1.707) |
| **Baseline blood eosinophil count category,* n (%), cells/µL**  <150 150–<300  300–<500  500–<700  ≥700 | n=144  8 (6)  12 (8)  30 (21)  33 (23)  61 (42) | n=173  17 (10)  11 (6)  54 (31)  28 (16)  63 (36) | n=260  16 (6)  20 (8)  63 (24)  56 (22)  105 (40) | n=57  9 (16)  3 (5)  21 (37)  5 (9)  19 (33) |
| **Allergic asthma, n (%)** | 76 (53) | 117 (67) | 158 (61) | 35 (60) |
| **Atopic sensitization, n (%)** | n=144  57 (40) | n=172  74 (43) | n=259  108 (42) | n=57  23 (40) |
| **Baseline ACT score, mean (SD)** | n=136  14.4 (5.03) | n=143  13.8 (5.05) | n=244  14.2 (5.02) | n=35  13.3 (5.19) |
| **Baseline ACT score category, n (%)**  <20 (uncontrolled)  ≥20 (controlled) | n=136  115 (85)  21 (15) | n=143  122 (85)  21 (15) | n=244  208 (85)  36 (15) | n=35  29 (83)  6 (17) |
| **Baseline post-bronchodilator FEV_1_%pred, mean (SD)** | n=129  76.2 (23.89) | n=80  76.5 (22.11) | n=172  77.1 (23.24) | n=37  72.9 (22.82) |
| **OCS dependent in the 12 months pre-mepolizumab treatment, n (%)** | 60 (42) | 72 (41) | 105 (40) | 27 (47) |
| **Baseline OCS dose, median (IQR), mg/day** | n=45  10.0 (5.0–15.0) | n=53  8.0 (5.0–20.0) | n=80  9.2 (4.8–16.8) | n=18  10.3 (5.0–20.0) |
| **Previous omalizumab treatment, n (%)** | n=144 52 (36) | n=173  69 (40) | n=259  105 (41) | n=58  16 (28) |
| **Comorbidities, n (%)**  Anxiety  Atopic dermatitis  Bronchiectasis  Depression  EGPA  GERD  Hypersensitivity to NSAIDs  CRSwNP | 33 (23)  2 (1)  36 (25)  29 (20)  6 (4)  35 (24)  19 (13)  64 (44) | 29 (17)  2 (1)  23 (13)  26 (15)  5 (3)  32 (18)  23 (13)  83 (48) | 51 (20)  2 (<1)  52 (20)  47 (18)  9 (3)  58 (22)  37 (14)  121 (47) | 11 (19)  2 (3)  7 (12)  8 (14)  2 (3)  9 (16)  5 (9)  26 (45) |

*Where a blood eosinophil count of zero was recorded, a small value (i.e., minimum all non-missing results/2) was added prior to log transformation.

ACT, asthma control test; BMI, body mass index; CRSwNP, chronic rhinosinusitis with nasal polyps; EGPA, eosinophilic granulomatosis with polyangiitis; FEV_1_, forced expiratory volume in 1 second; GERD, gastroesophageal reflux disease; IQR, interquartile range; NSAID, non-steroidal anti-inflammatory drug; OCS, oral corticosteroid; pred, predicted; SD, standard deviation.
