# Supplementary figures and images for: Remission outcomes in severe eosinophilic asthma with mepolizumab therapy: Analysis of the REDES study
Source: Front Immunol. 2023 Apr 12;14:1150162. doi: 10.3389/fimmu.2023.1150162 (PMC10131245; doi:10.3389/fimmu.2023.1150162)

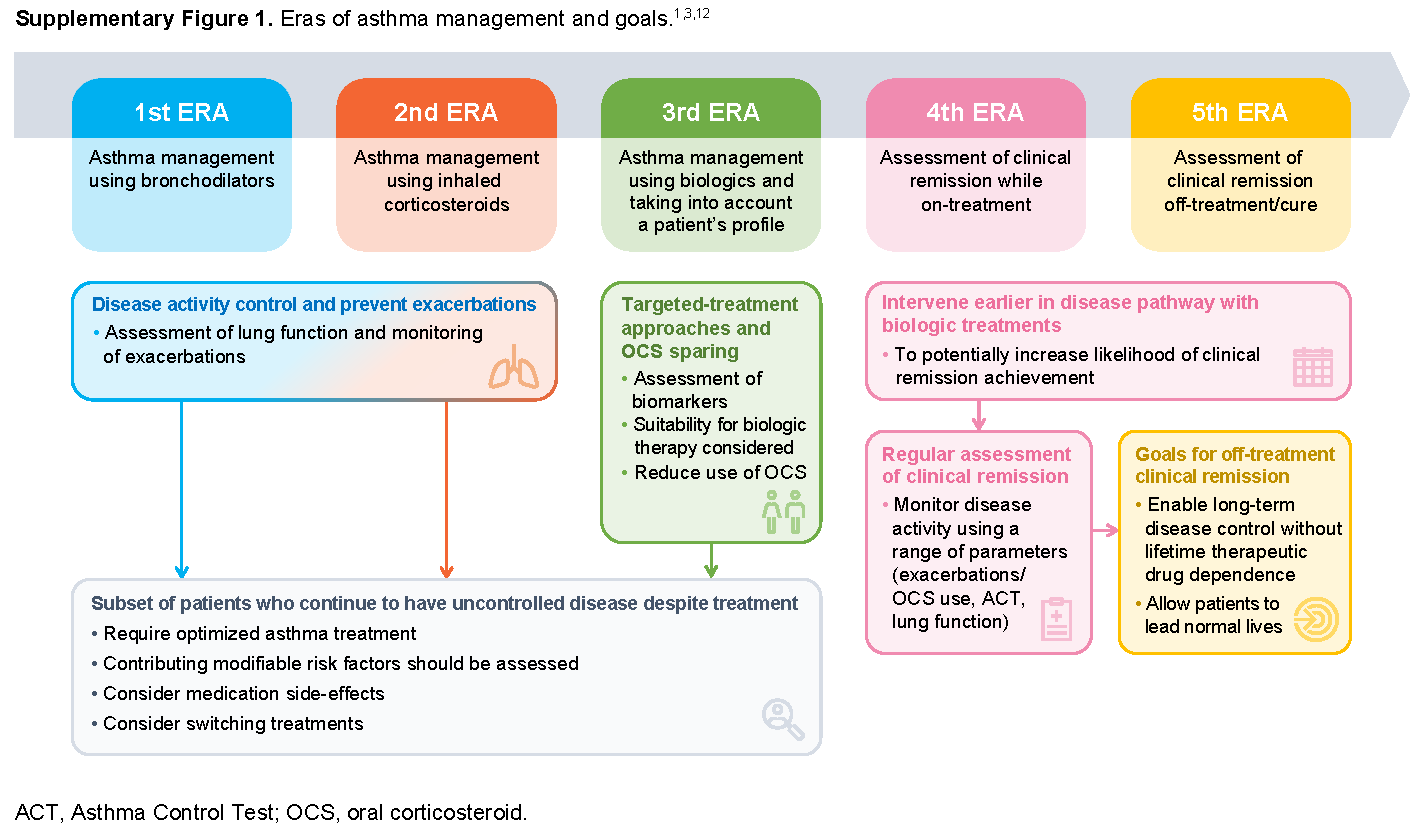

Supplement: Supplementary file 2 [file Image_1.tif]

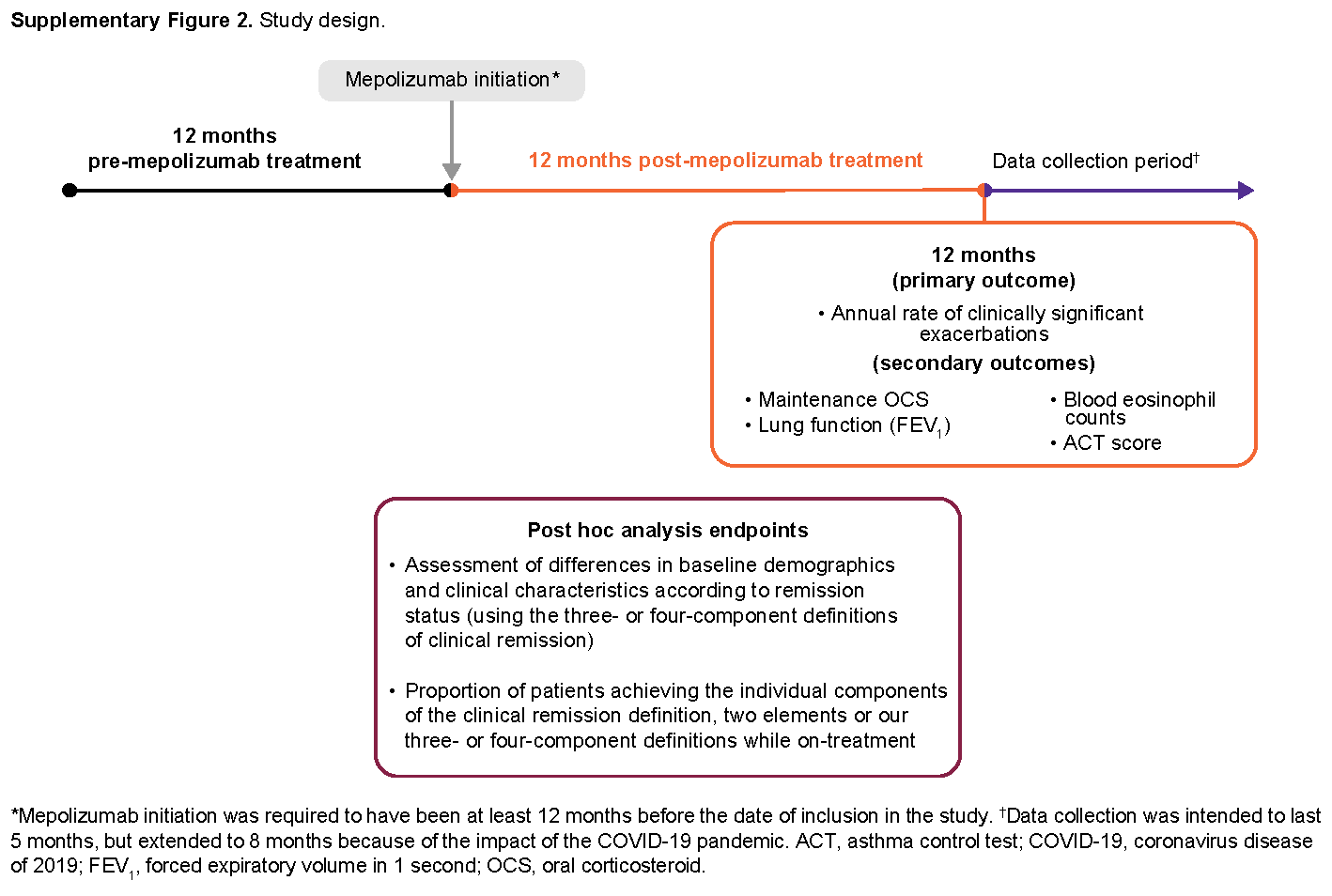

Supplement: Supplementary file 3 [file Image_2.tif]
